# Editorial: Natural products in the fight against antibiotic resistance: addressing the WHO priority pathogens

**DOI:** 10.3389/fphar.2026.1845222

**Published:** 2026-04-27

**Authors:** Tushar Dhanani, Maryam Shafaati, Sushmita Nath, John Thor Arnason

**Affiliations:** 1 Center for Viticulture and Small Fruits Research, College of Agriculture and Food Science, Florida Agricultural and Mechanical University, Tallahassee, FL, United States; 2 Research Center for Antibiotic Stewardship and Antimicrobial Resistance, Infectious Diseases Department, Imam Khomeini Hospital Complex, Tehran University of Medical Sciences, Tehran, Iran; 3 Center for Communicable Disease Control, Ministry of Health and Medical Education, Tehran, Iran; 4 University Hospital of Münster, University of Münster, Münster, Germany; 5 Department of Biology, University of Ottawa, Ottawa, ON, Canada

**Keywords:** antimicrobial resistance (AMR), biofilm inhibition, drug discovery, natural products, WHO priority pathogens

**FIGURE 1:**
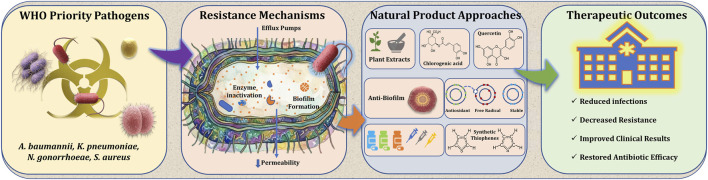
Overview of resistance mechanisms in WHO priority pathogens and corresponding natural product-based intervention strategies.

Antimicrobial resistance (AMR) continues to escalate as a global health crisis, driven by the rapid emergence of multidrug-resistant (MDR) pathogens and the diminishing efficacy of existing antibiotics. The World Health Organization (WHO) priority pathogen list has provided a critical framework to guide research and therapeutic development; however, progress in addressing these pathogens remains uneven. This Research Topic brings together studies examining the scientific and translational landscape of antimicrobial discovery, with particular emphasis on natural products and related strategies targeting WHO-priority bacteria, as summarized in Figure 1.

A fundamental perspective was provided by Abdallah et al., who synthesized current knowledge on resistance mechanisms across WHO-priority pathogens. This work highlights key molecular drivers, including reduced membrane permeability, efflux pump overexpression, enzymatic drug inactivation, target modification, biofilm formation, and horizontal gene transfer. It also emphasizes the limitations of traditional antibiotic discovery pipelines and the growing clinical burden of resistant infections. Emerging strategies discussed include artificial intelligence-assisted drug discovery, antimicrobial peptides, phage therapy, CRISPR-based approaches, and resistance-modifying combinations, with natural product-derived compounds identified as an important component of future solutions.

Complementing this mechanistic study, Robledo Almonacid et al. evaluated the global scientific response to the WHO priority pathogen framework using an artificial intelligence-based tool (LLMzCor). Analysis of publication trends before and after the WHO alert demonstrated only modest increases in research focused on new treatments and minimal changes in immunization strategies. These findings underscore a persistent gap between global prioritization efforts and measurable scientific output, reinforcing the need for more coordinated research initiatives.

Several contributions explored natural products as antimicrobial agents against specific WHO-priority pathogens. Liao et al. investigated 13 traditional Chinese medicine (TCM) preparations against *Neisseria gonorrhoeae*, demonstrating significant antibacterial activity in selected formulations, including Coptidis Rhizoma, Phellodendri Chinensis Cortex, Forsythiae Fructus, Taraxaci Herba, and Scutellariae Radix. The lack of correlation between minimum inhibitory concentrations (MICs) of several TCMs and conventional antibiotics suggests distinct mechanisms of action and a reduced likelihood of cross-resistance.

The role of natural products as adjunctive therapies was further illustrated by Li et al. in a meta-analysis evaluating Xuebijing (XBJ) for the treatment of *Acinetobacter baumannii* infections. Integration of clinical data from randomized controlled trials with network pharmacology and molecular modeling demonstrated improved clinical outcomes, enhanced bacterial clearance, and reduced inflammatory markers in patients receiving XBJ alongside conventional therapy. Active components such as scutellarin and salvianolic acids were identified as modulators of inflammatory pathways, suggesting host-directed therapeutic effects.

At the interface of host–pathogen interactions and resistance dissemination, Wang et al. examined the effects of chlorogenic acid on carbapenem-resistant *Klebsiella pneumoniae*. The findings showed that chlorogenic acid attenuated virulence by inhibiting macrophage pyroptosis and disrupted the transfer of resistance genes mediated by outer membrane vesicles, highlighting a dual-action strategy beyond conventional bactericidal mechanisms.

The potential of natural compounds to enhance existing antibiotic therapies was also explored by Bustos et al. through the study of flavonoids such as quercetin and luteolin. These compounds mitigated antibiotic-induced oxidative stress in host cells while maintaining antimicrobial activity. In particular, luteolin demonstrated synergistic effects with antibiotics against *Staphylococcus aureus*, supporting the feasibility of combination strategies that improve therapeutic outcomes while reducing host toxicity.

A systematic evaluation of specific natural compounds was provided by Farhadi et al. in a review of thymol and carvacrol against *Klebsiella* species. The compiled evidence demonstrated consistent antibacterial and anti-biofilm activity, with predominantly synergistic interactions when combined with conventional antibiotics, reinforcing the importance of targeting biofilm-associated resistance.

Beyond plant-derived compounds, this Research Topic also included studies focused on synthetic and semi-synthetic antimicrobial agents inspired by natural scaffolds. Molina-Panadero et al. identified novel thiophene derivatives with activity against colistin-resistant *Acinetobacter baumannii* and *Escherichia coli*. These compounds exhibited bactericidal activity, increased membrane permeability, and reduced bacterial adherence, demonstrating how chemical innovation can complement natural product discovery in addressing resistant Gram-negative pathogens.

Finally, a broader conceptual perspective was offered by Dyson et al. through a review of microbial defense systems as a source of new antimicrobial strategies. By examining the molecular ecology of antibiotic production and microbial competition, this work emphasizes the need to move beyond traditional paradigms of drug discovery and advocates for sustainable approaches integrating ecological and evolutionary insights to mitigate future resistance cycles.

Collectively, the contributions in this Research Topic illustrate the multifaceted role of natural products and related strategies in addressing antimicrobial resistance. From direct antimicrobial activity and synergistic combinations to modulation of host responses and inhibition of resistance transmission, these studies underscore the versatility of natural compounds in tackling WHO-priority pathogens. Despite encouraging advances, several critical limitations continue to constrain the clinical translation of natural product-based antimicrobial strategies. Variability in chemical composition due to differences in biological sources, cultivation conditions, and extraction methods can affect reproducibility and therapeutic consistency. Challenges in standardization, quality control, and regulatory alignment remain significant barriers to widespread clinical adoption. Limited pharmacokinetic and toxicological characterization, together with the scarcity of large, well-controlled clinical trials, further restrict the translation of promising laboratory findings into approved therapeutic interventions.

Addressing these challenges will require clearly defined research priorities and coordinated multidisciplinary efforts. Future studies should focus on standardized protocols for natural product characterization, rigorous preclinical validation using clinically relevant infection models, and the design of multicenter clinical trials to establish efficacy and safety across diverse patient populations. Integration of advanced analytical technologies, systems biology, and artificial intelligence-driven discovery platforms may accelerate the identification and optimization of next-generation antimicrobial agents. Strengthening collaboration among academic researchers, clinicians, industry partners, and regulatory agencies will be essential to bridge the gap between discovery and implementation.

Taken together, this body of work reinforces the importance of integrating natural product research with advances in computational tools, systems biology, and clinical sciences. By systematically addressing translational barriers and prioritizing reproducibility, standardization, and clinical validation, the field can progress toward the development of safe, effective, and scalable antimicrobial interventions capable of responding to the evolving global threat of antimicrobial resistance. The editors sincerely thank all authors and reviewers for their valuable contributions to this Research Topic.

